# The Use of Triaxial Accelerometers and Machine Learning Algorithms for Behavioural Identification in Domestic Cats (*Felis catus*): A Validation Study

**DOI:** 10.3390/s23167165

**Published:** 2023-08-14

**Authors:** Michelle Smit, Seer J. Ikurior, Rene A. Corner-Thomas, Christopher J. Andrews, Ina Draganova, David G. Thomas

**Affiliations:** School of Agriculture and Environment, Massey University, Palmerston North 4410, New Zealand

**Keywords:** domestic cat, accelerometer, random forest, self-organizing map, behaviour classification

## Abstract

Animal behaviour can be an indicator of health and welfare. Monitoring behaviour through visual observation is labour-intensive and there is a risk of missing infrequent behaviours. Twelve healthy domestic shorthair cats were fitted with triaxial accelerometers mounted on a collar and harness. Over seven days, accelerometer and video footage were collected simultaneously. Identifier variables (n = 32) were calculated from the accelerometer data and summarized into 1 s epochs. Twenty-four behaviours were annotated from the video recordings and aligned with the summarised accelerometer data. Models were created using random forest (RF) and supervised self-organizing map (SOM) machine learning techniques for each mounting location. Multiple modelling rounds were run to select and merge behaviours based on performance values. All models were then tested on a validation accelerometer dataset from the same twelve cats to identify behaviours. The frequency of behaviours was calculated and compared using Dirichlet regression. Despite the SOM models having higher Kappa (>95%) and overall accuracy (>95%) compared with the RF models (64–76% and 70–86%, respectively), the RF models predicted behaviours more consistently between mounting locations. These results indicate that triaxial accelerometers can identify cat specific behaviours.

## 1. Introduction

Animal behaviour can provide a reliable and non-invasive indication of animal health and welfare. In domestic cats (*Felis catus*), behavioural changes can indicate the presence of illness, pain, or distress [1]. Behavioural monitoring of pet cats is often carried out by their owner(s). However, owner observation is subjective, and they cannot monitor their pets continuously, and they may therefore miss early and subtle signs of illness. This is exacerbated by the fact that changes in behaviour in response to illness and/or pain can often be subtle and well disguised by the cat [1]. In addition, any behavioural research trial using traditional methods, scoring behaviour manually either directly or from video recordings, is labour-intensive. Accelerometers are a promising tool to help continuously monitor the behaviour of animals, including domestic cats.

To date, few studies in domestic cats have used accelerometer data from non-commercial triaxial accelerometers to distinguish between or identify specific behaviours [2,3]. Accelerometers can measure body movement in terms of acceleration in one to three orthogonal planes: craniocaudal (surge; forwards/backwards), mediolateral (sway; left/right), and dorsoventral (up/down) [4]. Measuring these accelerations in multiple directions allows detection of both dynamic (motion) and static (gravity) accelerations [4,5]. Watanabe et al. [3] were able to successfully distinguish drinking (100% accuracy), eating (68% accuracy), trotting (78% accuracy), and galloping (71% accuracy) in a single domestic cat using only acceleration data along the craniocaudal plane (forwards/backwards). Galea et al. [2] successfully built two identifying models for twelve different behaviours from triaxial acceleration data from ten domestic cats, using random forest (RF) and self-organizing maps (SOM).

The location of the accelerometer on the animal is an important factor to consider. Aspects to consider when determining the attachment site include the animal species, potential effects of attachment site on behaviour, behaviours that are of interest in the study, and rigidity of the attachment of the accelerometer on a site [4,6]. For example, an accelerometer attached to the back of an animal is less likely to register fine-scale head movements involved in eating behaviour which may be detectable by a collar-mounted accelerometer [4]. In domestic cats, the most commonly used site of accelerometer attachment is a collar with the device positioned ventrally. Attachment to a collar, however, can result in rotation of the accelerometer and residual movement (i.e., movement of the device after the physical movement stops) which is dependent on the looseness of the collar, weight of the accelerometer, and the animal’s behaviour [4,6]. A greater model accuracy was found in dogs for accelerometers attached to a harness than a collar [7]. It is therefore important to identify behaviours of interest before determining the accelerometer placement location.

To be able to identify behaviours using accelerometer data, machine learning (ML) techniques are often used to build classifier models. Depending on the dataset, some ML techniques might be a better fit than others. Nathan et al. [8] reported that the random forest (RF) ML technique had the highest accuracies for identifying behaviour of free ranging griffon vultures from accelerometer data. In that study, the accuracies of five frequently used supervised ML techniques were compared: linear discriminant analysis (LDA), support vector machines (SVM), classification and regression trees (CART), random forests (RF), and an artificial neural network (ANN) [8]. Galea et al. [2] compared SOM with RF and found that SOM had a higher overall accuracy than RF (99.6% vs. 98.9%, respectively) for behaviour classification of domestic cats wearing a harness-mounted accelerometer.

In the present study, commercially available accelerometers were attached to domestic cats on both a collar and a harness, to capture movement data needed to build behaviour identifier models. The aim of the current study was to compare model performance between the two sites of attachment, (collar and harness) and two machine learning techniques (RF and supervised SOM). It was hypothesised that both RF and SOM models of harness-mounted accelerometer data would have better overall performance, or ability to identify behaviour, when compared with models of the collar-mounted accelerometer, due to the greater rigidity of the harness-mounted accelerometer reducing the risk of individual and residual device movement. However, given that the collar-mounted accelerometer was expected to be more likely to detect finer-scale head movements, it was expected that the model of the collar-mounted accelerometer would have higher accuracy in detecting eating behaviour than harness-mounted accelerometer models. Furthermore, as the SOM model had higher overall performance compared with the RF model in the study by Galea et al. [2], it was also hypothesised that in the current study SOM models would have higher overall performance compared with the RF model.

## 2. Materials and Methods

The study was conducted at the Massey University Centre for Feline Nutrition, Palmerston North, New Zealand (latitude 40°23′ S, longitude 175°36′ E) between 30 June and 7 July 2021. This study was approved by the Massey University Animal Ethics Committee (MUAEC 21/23).

### 2.1. Animals and Design

**Figure 1:**
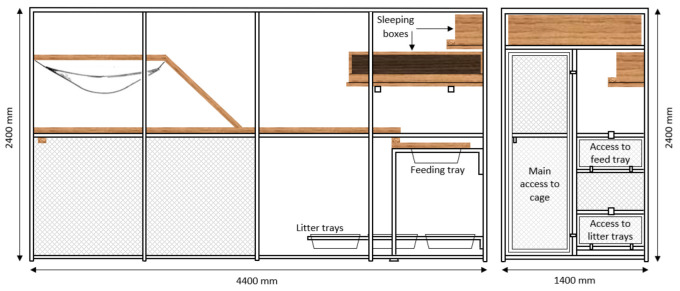
Colony cages as seen from the side (**left**) and front (**right**), measuring 1400 mm × 2400 mm × 4400 mm.

The study comprised two phases: habituation and data collection. For the habituation phase, 16 healthy desexed male (n = 7) and entire female (n = 9) domestic shorthair cats aged from 2.3 to 4.4 years (mean ± SD, 2.64 ± 0.62 years) and weighing between 2.6 to 5.2 kg (3.93 ± 0.89 kg) were assessed for inclusion in the study. The habituation phase lasted for five weeks: four weeks for habituation to the cat harness and one week for habituation to the accelerometers attached to both a collar and harness. Cats were already accustomed to wearing collars and thus relatively little habituation was needed for accelerometer attachment. However, three cats did not habituate to the harness and one cat persisted in biting the harnesses of other cats, thus these animals were removed from the study. A subset of twelve desexed male (n = 5) and entire female (n = 7) cats aged from 2.3 to 4.4 years (mean ± SD, 2.75 ± 0.69 years) and weighing between 2.6 to 5.1 kg (3.85 ± 0.82 kg) who had successfully completed the habituation period were included in the data collection phase. During data collection, each cat wore two accelerometers, one attached to a collar and one to a harness, for seven consecutive days. During the same seven days, cats were under continuous video surveillance. Throughout both phases of the study, cats were housed in two semi-outdoor colony cages (Figure 1), fed a complete and balanced [9] commercial canned diet (Heinz Wattie’s Ltd., Hastings, New Zealand), and had *ad libitum* access to water.

### 2.2. Data Collection

**Figure 2:**
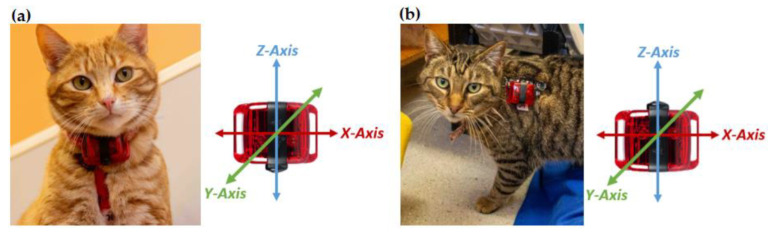
Placement and orientation of the ActiGraph wGT3X-BT accelerometer on a (**a**) collar and (**b**) harness.

Cats were fitted with a collar and harness to which an ActiGraph wGTX-BT accelerometer (weighing 19 g and measuring 33 mm × 46 mm × 15 mm) was attached (ActiGraph, Pensacola, FL, USA). On the collar the accelerometer was positioned ventrally (Figure 2a), and on the harness it rested on the left shoulder blade (Figure 2b). The orientation of the accelerometers was uniform across all cats for each mounting location. For the collar-mounted accelerometer, the orientation of the *X*, *Y*, and *Z* axes were lateral, dorso-ventral, and cranio-caudal, respectively, whereas for the harness-mounted accelerometer the orientation of the *X*, *Y*, and *Z* axes were cranio-caudal, dorso-ventral, and lateral, respectively. Acceleration data were sampled at a frequency of 30 Hz (raw acceleration data), with a dynamic range of ±8 g. For each cat, a unique pattern of reflective tape was placed on the two accelerometers to allow cat identification under infrared light.

Cats were filmed in real time using a 4K Swann^®^ security camera system (Swann Communications USA, Santa Fe Springs, CA, USA) capable of automatically switching between natural and infrared light, enabling continuous observation under natural light and dark conditions. A selection of active (climbing, jumping, fighting, playing, rolling, rubbing, running, trotting, walking), inactive (lying, sitting, standing), maintenance (digging, drinking, eating, grooming, littering, scratching, shaking), and other (other, out of sight, allogrooming, human contact) behaviours were then retrospectively scored using BORIS [10] by a single scorer (Table 1). Behaviours were scored continuously and were then exported using a one-second (s) time interval. For all cats, behaviour was scored for the first day of data collection, between 09:00 AM and 02:00 PM, when cats were expected to show the largest range of behaviours because of the presence of staff and feeding within those hours.

### 2.3. Model Classification

All data computation and statistical analyses were carried out using RStudio version 4.1.1 [14]. The R code, including R packages used, has been published in a GitHub repository [15].

#### 2.3.1. Intra-Rater Reliability

To test the intra-rater reliability, a subset of five randomly selected 15 min video recordings were scored for behaviour for a second time, with a time interval of six months between the first and second behaviour scoring. Intra-rater reliability was tested using the Kappa coefficient (κ) with the R package ‘irr’ [16]. Results for the Kappa coefficient were interpreted according to Fleiss [17], where values > 0.75 indicated excellent agreement, 0.40 to 0.75 indicated fair to good agreement, and <0.40 indicated poor agreement.

#### 2.3.2. Derivation Identifier Variables

Raw acceleration data for each axis were exported from the devices using ActiLife software (version 6.13.4; ActiGraph, Pensacola, FL, USA). Using RStudio v1.4.1 [14], a total of 32 identifier variables were derived from the raw accelerometer data and summarized into 1 s epochs (i.e., time interval): mean acceleration (*X*, *Y*, *Z*), sum acceleration (*X*, *Y*, *Z*), minimum (min) acceleration (*X*, *Y*, *Z*), maximum (max) acceleration (*X*, *Y*, *Z*), standard deviation (sd) of acceleration (*X*, *Y*, *Z*), skewness (*X*, *Y*, *Z*), kurtosis (*X*, *Y*, *Z*), correlation (*XY*, *XZ*, *YZ*), vector magnitude (VM; mean, sum, min, max, sd, skewness, kurtosis), and overall dynamic body acceleration (ODBA; see Table 2 for detailed description of each identifier variable).

#### 2.3.3. Building Behaviour Identifier Models

Before building the models, behaviours that were not observed and behaviours classified as “other” or “out of sight”, were removed from the dataset. Two ML techniques were used to build identifier models to identify behaviours in domestic cats: (I) RF and (II) supervised SOM. RF is a ML technique that builds a multitude of decision trees and combines the output at the end [18]. SOM is a ML technique that produces two-dimensional maps, usually a grid of nodes, of multi-dimensional and complex data, where nodes that are similar are located close to each other [19]. A model was made with each modelling technique for each mounting location: RF for collar (CRF) and harness (HRF) and SOM for collar (CSOM) and harness (HSOM).

The RF models were built using the R packages ‘caret’ [20] and ‘randomForest’ [21]. The default settings for the number of trees (n = 500) and the number of variables randomly sampled as candidate at each split (n = $\sqrt{n_{variables}}$) were used. The SOM models were built using the R package ‘Kohonen’ (version 3.0.11) [22], with the number of times the dataset was presented to the network set to the default setting (n = 100). Each model was built using a subset (70%) of the complete dataset. The model was then used, and tested, to identify the behaviour using the identifier variables of the remaining 30% of the complete set. A confusion matrix was computed by comparing the identified behaviours with the observed behaviours.

#### 2.3.4. Model Evaluation

Using the computed confusion matrix, behaviours for each model were labelled as true positive (TP) when the behaviour was correctly identified by the model, true negative (TN) when the behaviour was correctly identified by the model as not occurring, false negative (FN) when the behaviour was observed but not identified by the model, or false positive (FP) when the behaviour was identified by the model but was not observed. Using data from the confusion matrix, the accuracy (Equation (1)), precision (Equation (2)), sensitivity (Equation (3)), and specificity (Equation (4)) were calculated for each behaviour:

Accuracy = (TP + TN)/(TP + TN + FP + FN),(1)

Precision = TP/(TP + FP),(2)

Sensitivity = TP/(TP + FN),(3)

Specificity = TN/(TN + FN).(4)

Overall model performance was determined by calculating the overall accuracy (Equation (5)) and Kappa coefficient (κ; Equation (6)) [23]. Results for the Kappa coefficient were interpreted according to Fleiss [17].

(5) Overallaccuracy=(TP+TN)/(TP+TN+FP+FN)

(6) $$\hat{\kappa }=\left(N\times \sum_{i=1}^{k}x_{ii}-\sum_{i=1}^{k}\left(x_{i+}\times x_{+i}\right)\right)/N^{2}-\sum_{i=1}^{k}\left(x_{i+}\times x_{+i}\right)$$

Multiple identifier models were built for both the collar- and harness-mounted accelerometer. For each model, the overall accuracy and estimated Kappa coefficient were evaluated, and for each behaviour the sensitivity, specificity, precision, and accuracy were calculated. Behaviours with low values were clustered with the lbehaviour they were misclassified as in the model if the behaviour belonged to the same category (active, inactive, maintenance), or were removed if in a different category. The new model was then built, and the overall accuracy and estimated Kappa coefficient were compared with the previous model. This behavioural selection and clustering, hereafter referred to as the ‘modelling round’, continued until the overall accuracy and Kappa coefficient did not improve or until only three behavioural categories remained: active, inactive, and maintenance.

### 2.4. Daily Activity Budgets and Dirichlet Regression

For accelerometer data not used to build the models, the behaviour of the cats was determined using the final models. Daily activity budgets (proportion of time spent showing each behaviour) were calculated for each cat. Using the R package DirichletReg [24], a Dirichlet regression with log link was performed as a function of mounting location (collar and harness), modelling technique (RF and SOM), and the day of observation. A Dirichlet regression allows for statistical testing between proportions [25]. The Dirichlet regression was performed separately for each modelling round (MR).

## 3. Results

From the 7 days of video footage from the 12 cats, a total of 166,754 s (≈46 h and 20 min; 3 h and 50 min per individual) of recordings were scored for behaviours. Of the 24 behaviours (Table 1) scored in the video recordings, 4 behaviours (‘rolling’, ‘running’, ‘drinking’, and ‘human contact’) were not observed at any time and were therefore removed before model building (Appendix A). Similarly, ‘fighting’ (n = 10 s) and ‘playing’ (n = 1 s) were removed due to their low occurrence. ‘Jumping horizontal’ (n = 53 s) and ‘jumping vertical’ (n = 186 s) were grouped into a single category ‘jumping’ (Appendix A). Seconds (i.e., datapoints) where cats were identified as ‘out of sight’ (n = 38,395 s) and ‘other’ (n = 4116 s), were also removed from the dataset. These changes resulted in a total of 124,230 datapoints which consisted of 15 different behaviours. Four modelling rounds were conducted which resulted in a total of 16 different behavioural identification models (Appendix A).

### 3.1. Intra-Rater Reliability

The intra-rater reliability, comparing the agreement between the first and second rounds of behaviour scoring of video data by the scorer, was tested and found to be excellent (κ = 0.93, *p* < 0.001).

### 3.2. Model Performance

It was observed that the initial models were overfitted to behaviours with large numbers of datapoints (e.g., lying; Appendix A), therefore behaviours with large numbers of datapoints were limited to n = 7000. For this reason, the numbers of datapoints for inactive and maintenance were also limited to n = 5000 for the fourth and final round of modelling (Appendix A). Datapoints were limited by randomly selecting 7000 or 5000 datapoints for each behaviour using the sample function in R.

The first modelling round included 15 behaviours which included climbing, jumping, rubbing, trotting, walking, lying, sitting, standing, grooming, littering, digging, eating, scratching, shaking, and allogrooming. In the first modelling round, the confusion matrices showed that some behaviours were not identified by the models (true positive = 0), e.g., climbing, digging, and allogrooming using the CRF model (Supplementary Material Tables S1–S4). Climbing, jumping, rubbing, digging, shaking, and allogrooming were removed before the second modelling round because of their small sample size. Trotting was often misclassified as walking in both the CRF and HRF models of the first modelling round, and the two were therefore merged into “active” (Supplementary Material Tables S1 and S2). Scratching was often misclassified as grooming in both the CRF and HRF models of the second modelling round and was merged with grooming before the third modelling round (Supplementary Material Tables S5 and S6). The remaining models identified all of the included behaviours (Supplementary Material Tables S9–S16) with an accuracy ≥0.75 (Supplementary Material S2 Tables S17–S20). The precision with which the models identified each behaviour ranged from 0.25 to 1.00; however, precision could not be calculated for behaviours that were not identified by the models (Supplementary Material S3 Tables S21–S24). The sensitivity of the models that contained behaviours that could not be identified was 0.00 (Supplementary Material S4 Tables S25–S28). For behaviours that were identified by the models, sensitivity ranged from 0.02 to 1.00. Precision and sensitivity decreased as the number of false positives or false negatives, respectively, increased compared with the number of true positives. Model specificities for identified behaviours were ≥0.90, except for eating in the RF models in the third modelling round, where the specificity was 0.72 for the CRF model, and 0.76 for the HRF model (Supplementary Material S5 Tables S29–S32).

Irrespective of mounting location, the overall performance values of the SOM models were higher than those of the RF models, with both Kappa and overall accuracy values > 0.95 for the SOM models (Table 3). The RF models generally showed fair to good agreement (κ between 0.40 and 0.75) between the observed and identified behaviours. The harness HRF models showed excellent agreement (κ > 0.75) in the second and third modelling round (Table 3). For the RF models, both the Kappa and overall accuracy values were higher for the HRF models than the collar CRF models in modelling rounds 1–3 (Table 3). It was only in the fourth modelling round, containing only three behavioural categories, that the CRF models outperformed the HRF models (Table 3). The overall performance values for the CSOM and HSOM models were very similar, apart from modelling round three, where the overall performance values for the HSOM model were higher than those of the CSOM model (Table 3).

### 3.3. Daily Activity Budgets

Accelerometer data that were not used to build the models (a total of six days) were used to identify the behaviour of each cat using each model in each modelling round. For each model, the proportion of time spent showing each identified behaviour was compared using Dirichlet regression [25].

Models from the first modelling round had low precision and sensitivity for identifying behaviours due to very low or zero true positives (climbing, jumping, trotting, digging, shaking, allogrooming) due to either not being identified or their very low occurrence (Table 4). Behaviours with high values overall for performance, including walking, lying, sitting, standing, grooming, eating, had varying results, depending on the model. The most frequently identified behaviours were lying (28.20–52.69%) and sitting (18.74–28.93%; Table 4). Grooming ranged from as low as 6.99% to as high as 20.69% and eating ranged from 3.32% to 22.67% (Table 4). For the RF models, differences in mean daily percentages were found for lying (*p* < 0.001), littering (*p* = 0.017), and eating (*p* = 0.005) between the CRF and HRF models (Table 4). For SOM models, differences were found in walking, lying, sitting, standing, and grooming (*p* ≤ 0.001 for all; Table 4) between the CSOM and HSOM models. Comparing the modelling techniques for the collar models (CRF and CSOM), the only behaviour that did not show a difference was rubbing (*p* = 0.104; Table 4). Comparing the modelling techniques for the harness models (HRF and HSOM), no differences were found for rubbing (*p* = 0.472) and walking (*p* = 0.737; Table 4). Most behaviours that showed no differences were behaviours with low precision and sensitivity (Supplementary Material S3 Tables S21–S24 and Supplementary Material S4 Tables S25–S28).

Despite the high results for performance of littering in both the CSOM and HSOM models and scratching in the HSOM model, these behaviours were not predicted at all in the second modelling round, but were identified by both the CRF and HRF models (Table 5). Lying (29.17–52.52%) and sitting (17.47–28.57%) remained the most commonly identified behaviours in the second modelling round (Table 5). Eating (3.22–22.15%) and grooming (7.15–18.91%) behaviours showed a wide range depending on the model used, which was similar to the first modelling round (Table 5). For the second modelling round, differences in mean daily percentages were found between the CRF and HRF models for lying (*p* < 0.001) and eating (*p* = 0.030; Table 5). The identified percentages of behaviours between the CSOM and HSOM were different (*p* < 0.05) for all behaviours that were identified by the models (Table 5). The CRF and CSOM models showed differences for active (*p* = 0.041), lying (*p* > 0.001), sitting (*p* < 0.001), standing (*p* < 0.001), grooming (*p* = 0.019), and eating (*p* = 0.004; Table 5). The HRF and HSOM models showed differences for active (*p* = 0.025), lying (*p* < 0.001), sitting (*p* < 0.001), grooming (*p* = 0.003), and eating (*p* < 0.001; Table 5).

The most frequently identified behaviours in the third modelling round remained lying (27.82–53.21%) and sitting (20.66–28.04%), with grooming behaviour ranging from 7.59% to 13.91% (Table 6). As seen in the second modelling round, eating behaviour showed a large range (3.28% to 26.79%; Table 6). In the third modelling round, no differences in the mean percentages of identified behaviours were found between the CRF and HRF models (*p* > 0.05), whereas all behaviours differed between the CSOM and HSOM models (eating *p* = 0.012, all other behaviours *p* < 0.001; Table 6). Differences in mean daily percentages were found between the CRF and CSOM models for active, lying, sitting, standing, grooming (all *p* < 0.001), and eating (*p* = 0.002; Table 6). For the HRF and HSOM models, differences were found for active, lying, and sitting (all *p* < 0.001), and eating (*p* = 0.011; Table 6).

For the fourth and final modelling round, the behaviours present in the third modelling round were merged into three main categories: active, inactive, and maintenance. Inactive behaviour was the most commonly identified behavioural category (51.16–84.46%), followed by maintenance (10.90–34.06%) and active (3.64–14.78%; Table 7). In the fourth modelling round, no differences were found in mean daily percentages of active (*p* = 0.898), inactive (*p* = 0.574), and maintenance (*p* = 0.907) behaviours between the CRF and HRF models (Table 7). For the SOM models, there was a difference in mean daily percentage between the CSOM and HSOM models for inactive behaviours (*p* < 0.001; Table 7). Differences in mean daily percentages were found between the CRF and CSOM for all three categories, which was also true between the HRF and HSOM (all *p* < 0.001; Table 7).

The effect of day was included in the Dirichlet regression and showed that, in the first modelling round, day influenced the proportion of walking (*p* < 0.001), lying (*p* = 0.004), sitting (*p* < 0.001), standing (*p* < 0.001), grooming (*p* < 0.001), and eating (*p* < 0.001) behaviour (Table 4). In the second modelling round, day influenced active (*p* = 0.019), sitting (*p* < 0.001), standing (*p* = 0.020), grooming (*p* = 0.027), and eating (*p* = 0.019; Table 5). In the third modelling round, day influenced active (*p* = 0.017), lying (*p* = 0.025), sitting (*p* < 0.001), standing (*p* = 0.014), grooming (*p* = 0.004), and eating (*p* = 0.011) behaviour (Table 6). In modelling round four, active (*p* = 0.076), inactive (*p* = 0.766), and maintenance (*p* = 0.413) were not influenced by day.

## 4. Discussion

The current study aimed to build a model to identify cat behaviour using accelerometer data. Generally, the more classes that were included in a model, the lower the accuracy of it. In the current study, the models for three behavioural classes (active, inactive, and maintenance) had the highest accuracy (Table 3). In the current study, similar behaviours that were often misclassified were merged (e.g., trotting and walking) or removed from the model (e.g., digging). This reduction of behaviour classes lowers the risk of misclassification, resulting in fewer false positives and false negatives, thereby increasing the accuracy. Similar results were found in a study on cheetahs (*Acinonyx jubatus*), where merging of similar behaviours resulted in models with higher accuracy [13]. Another study, modelling behaviour using accelerometer data of oystercatchers (*Haematopus ostralegus*), found that a model with three behaviour classes had a lower absolute cross-validation error than a model with eight behaviour classes [26]. These results show that there is a trade-off between a more representative model, including more behaviours, and accuracy. Despite decreasing accuracy with increasing classes, all models in the current study had an accuracy ≥0.70.

Two modelling techniques, RF and SOM, and two mounting locations, collar and harness, were compared. Early models (data not shown) were able to identify behaviours that had relatively large sample sizes (e.g., lying), but had difficulty with behaviours with a sample size lower than 500 (e.g., climbing). In order to balance the dataset from which the models were built, behaviours with a large sample size were limited to 7000 datapoints. Despite this limit, the models from the first round of modelling had difficulty identifying behaviours with a low sample size such as climbing (n = 32), jumping (n = 239), and trotting (n = 308). Galea et al. [2] reported that SOM models of behaviours with a sample size less than 2000 datapoints had lower accuracy, sensitivity, and precision compared with behaviours with a sample size larger than 2000. Low sample sizes were the result of a behaviour being displayed infrequently.

In the current study, most behaviours with low sensitivity and precision were those that consisted of swift movements and/or behaviours of short duration (<one second). This is in agreement with [27] who reported that models of data derived from collar-mounted accelerometers attached to captive dingoes (*Canis lupus dingo*) had more difficulty identifying active behaviours consisting of swift movements performed over a short period of time. Capturing swift and short duration movements is a challenge especially encountered in small animal species, as movements are generally quicker than in larger animals [4]. A study using data from accelerometers attached to chipmunks (*Tamias*), a small-bodied animal (<100 g), found the lowest sampling frequency that resulted in negligible decrease in accuracy was 20 Hz [28]. In the current study, jumping was often misidentified as walking, a behaviour that was often observed and identified immediately before and after the jump. In the current study, the shortest lasting behaviour (jumping) lasted for an average of 0.89 s (results not shown). Accelerometer data were collected at 30 Hz and summarised into one-second epochs, which could have led to the acceleration signature of very short-lasting behaviours being lost in the signature of the behaviour immediately before or after they occurred. Choosing an epoch length that resembled the length of these shorter behaviours might help in the ability of models to identify them more accurately. In addition, a shorter epoch could also increase the sample size of infrequent behaviours. While a smaller epoch length can increase the sample size, it will increase the computational time to train and test the models [27]. In the current study, the total number of observations (Appendix A) for each short-lasting behaviour was very close to the total amount of time it was observed in the video recordings. A one-second epoch is therefore thought to be small enough when considering the behaviours included in the current study.

It was hypothesised that the harness-mounted accelerometer would have better performance values compared with the collar. There was little difference in Kappa and overall accuracy between the CSOM and HSOM models within each modelling round. However, the majority of the RF models had higher performance values when data from devices attached to a harness were compared with the collar. These results are in agreement with a study with domestic dogs, where harness-mounted accelerometers resulted in models where accuracy was higher (87–91%) than collar-mounted accelerometers (69–76%) [7]. One of the reasons cited for the higher accuracy of harness-mounted accelerometers compared with collar-mounted ones was the firmer attachment the harness provided for the device [7]. Collar attachment, in contrast, resulted in changes in device orientation and residual movement [7]. For cats, it is challenging to obtain a firmer device attachment with a collar, as securing the collar tightly might result in discomfort or injury [29]. Despite the harness models generally having better performance compared with the collar models when considering the RF modelling technique, the collar and harness models identified similar percentages of each behaviour within each behavioural round (Table 4, Table 5, Table 6 and Table 7).

In the current study, it was hypothesised that the models from the collar-mounted accelerometers would be better for identifying more subtle behaviours such as eating and drinking, as these behaviours are associated with head movement rather than body movement. While drinking and eating behaviours have been successfully distinguished using data from only one axis of an accelerometer attached to a collar on a single domestic cat [3], different attachment methods (harness vs. collar) have not been compared for these fine-scale behaviours in domestic cats. In the current study, the differences in performance values between the CSOM and HSOM models were very small, on average <1%. For the RF models, however, the performance values of the harness model for eating behaviour were always higher than those of the RF collar models. This could be the result of the design of the feeding area in the colony cages (Figure 1). Cats were often observed to place their front paws in the feeding tray, while their hind paws remained on the wooden walkway surrounding the feeding tray, resulting in a forward tilted posture where the head and shoulders were lower than their hips. This change in posture during eating is likely to have resulted in a greater change in the orientation of the harness-mounted accelerometer compared with the collar [7]. In a home situation it would be expected that food bowls would be on the ground or slightly elevated and thus will not result in the cat being tilted forward [30,31].

As hypothesised, in the current study the SOM models had higher Kappa and overall accuracy values compared with the RF models. These results are in agreement with previous findings reported on domestic cats by [2], where the SOM model had a mean accuracy of 99.6%, compared with the RF model of 98.9%. The mean accuracy of the RF model as reported by [2] was higher than the overall accuracy reported in the current study (70–83%). It should be noted, however, that mean accuracy and overall accuracy were calculated differently, and cannot therefore be directly compared.

In addition to Kappa and overall accuracy to determine model performance, the current study further evaluated the performance of the models. To date, rather than using models on a new dataset and then comparing the results, the ability of models to identify behaviours has been based on performance results, such as measures of accuracy [8,27]. In the current study, all models were used to identify the behaviour of the same 12 domestic cats, using accelerometer data that were not used in the training and testing of the model. The results showed that the identified behaviours, expressed as a percentage of total behaviour, were identified more consistently with the RF models between mounting locations and across modelling rounds than the SOM models.

A possible explanation for the seemingly poorer performance of the SOM models when presented with a new dataset is overfitting. Overfitting occurs when a model fits the training data so well, it memorises noise in the dataset, leading to a model that does not perform well on a new dataset [32]. There are different reasons why overfitting might occur, including a dataset that is too small and/or the presence of too many predictor variables, making the model overcomplicated [32,33]. Galea et al. [2] tested how the accuracy of the SOM changed with different sample sizes for the training dataset and reported that the accuracy of the SOM plateaued after 20,000 samples. With the exception of the fourth modelling round, the training dataset (70%) contained more than 20,000 samples, making the size of the dataset sufficient according to results reported by [2]. Since the sample size of the current study was probably sufficient, the most likely explanation for the overfitting of the SOM models is overcomplication of the models due to many predictor variables. Overfitting due to too many predictor variables can be avoided by either selecting fewer predictor variables, or by increasing the sample size [32]. The predictor variables included in the current study were based on the 26 predictor variables identified as most effective by [27] and the 31 used by [2]. The SOM models were simplified to as few as four predictor variables (mean for X, Y and Z, and ODBA), but this did not improve the results (results not shown).

A supervised SOM is a type of artificial neural network that consists of an input layer, a single hidden layer, and an output layer in the form of a grid map consisting of neurons [34]. There is a neighbourhood relationship between the neurons on the output grid map [34]. The neurons of the hidden layer are connected to the samples in the input layer through weights, and weights are updated with each iteration [35]. Due to the neighbourhood relationship between neurons, changes in the weight of one neuron will affect other neurons in its neighbourhood [34]. Allende et al. [36] reported that SOMs are sensitive to outliers because of how the changing weight of one neuron affects the neighbouring neurons. It is reasonable to expect outliers in behavioural datasets, as behaviour can be very dynamic. A cat sitting, for example, can sit completely stationary, but it might suddenly turn its head back when it hears a noise, triggering the accelerometer due to the movement. In both cases, the cat is identified simply as sitting but will result in different accelerometer traces where one might be identified as an outlier. It is possible that the outliers of the dataset not used to build the model included outliers that were different than those contained in the training dataset. This could have led to the SOM being able to identify behaviours in the training dataset with high accuracy but having problems with identifying behaviours in a novel dataset. The SOM models could possibly be improved by distinguishing more subtle differences in behaviour and posture, e.g., separately annotating sitting completely stationary and moving the head around while sitting. It was decided not to re-watch the video recordings to separately identify more subtle differences within a single behaviour, as scoring behaviour is very labour-intensive, and the results show that the RF models identify behaviours consistently across mounting locations and are the best fit for the dataset generated in this study.

Literature on the activity budget of domestic cats is limited and results cannot be compared directly due to differences in housing conditions, behaviours, and sampling method. Despite these differences, the time domestic cats spent on eating behaviours was surprisingly consistent, with daily time spent eating being approximately 2–3% [37,38,39,40]. The RF models showed that in the current study cats spent between ~3% and ~4% of their time eating, whereas this ranged between ~13% and ~28% when identified with the SOM models. Not only did the RF models identify behaviour more consistently between mounting locations, but they also identified eating behaviour with a similar percentage as reported in the literature. This further supports the choice of RF models for the dataset generated in the current study.

The present study provided an insight into the activity budgets of colony-housed cats. Irrespective of modelling technique used or mounting location, cats spent the majority of their time inactive. The RF models identified that inactive behaviours accounted for between ~83% and ~86% of all behaviours. This was greater than the ~62% observed in farm cats [40], but comparable to that observed in privately owned cats (~84%) [39]. The difference in the percentage of inactive behaviours between these studies is probably due to the farm cats having the ability to roam around the farm and hunt for prey, whereas the cats in the current study had limited opportunity to roam and had a consistent *ad libitum* food source. Panaman [40] reported that four of the five farm cats were active hunters, which resulted in active behaviours (hunting and travelling) being observed for ~18% of their day, whereas the colony-housed cats were active (walking, trotting) for only ~3–5% of their time when the RF models were used.

## 5. Conclusions

The current study successfully produced identification models using accelerometer data to identify cat behaviour, paving the way to make behavioural studies in domestic cats less labour-intensive in the future. A trade-off was found between the number of behaviours included in the model and accuracy; however, the accuracy for all models, including the models with 15 behaviours, was ≥0.70. Despite the higher performance values of the SOM models, the RF models appear to predict cat behaviour more consistently. No differences in performance values between the collar and harness were found. Performance values, especially sensitivity and precision, of less frequent behaviours can be improved by increasing the sample size in future studies. The current study also provided valuable information about activity budgets of colony-housed cats which spent most of their time inactive. More research is advised to examine the potential for accelerometers and machine learning to monitor behavioural changes in cats to allow health monitoring.

## Figures and Tables

**Table 1:** Ethogram including definitions for scored behaviours.

Behaviour	Description
***Active***	
Climbing [11]	Cat ascends and/or descends a vertical object or structure.
Jumping horizontal	Cat leaps from one point to another horizontally.
Jumping vertical	Cat leaps from one point to another vertically.
Fighting [12]	Cat engages in aggressive physical combat with another cat. Piloerection generally present of tail and back fur.
Playing [12]	Cat interacts with a (cat) toy or with another cat in a non-aggressive manner. Ears generally point forward, inverted U-shape of tail. Piloerection generally absent.
Rolling [11]	Cat rotates its body from side to side while lying on a horizontal surface. During the roll, when the cat is on its back, the belly is exposed, and all paws are in the air.
Rubbing	Cat rubs body against a surface or object.
Running [12,13]	Forward locomotion at a fast gait. Four-beat, asymmetric gait. Has a suspension phase. Fastest gait.
Trotting [12,13]	Forward locomotion at a swift gait performed. Two-beat, symmetric movement. Body is supported by two diagonal legs during contact with ground. Intermediate gate.
Walking [12,13]	Forward locomotion at a slow gait. Four-beat, symmetric movement with limbs moving sequentially. Includes slow walk (three or four feet contact ground during any one phase), fast walk (two or three feet contact ground during any one phase). Slowest gait.
***Inactive***	
Lying [11]	Body of the cat is in a horizontal position on a horizontal surface. Cat can be lying on its side, back, belly, or curled up.
Sitting [11]	Cat is in an upright position, with the hind legs flexed and under the body, and with front legs extended and straight at the front of the body.
Standing [11]	Cat is immobile and supporting the body with extended legs and all paws touching the surface.
***Maintenance***	
Digging [11]	Cat breaks up or moves substrate around with either one of its front paws.
Drinking [11]	Cat ingests water by lapping up with the tongue.
Eating [11]	Cat ingests food by chewing with the teeth and swallowing.
Grooming [11]	Cat cleans itself by either licking, scratching, biting, or chewing the fur on its body. Includes the licking of a front paw and wiping it over its own head.
Littering	Cat urinates or defecates.
Scratching [11]	Cat scratches its body using the claws of its hind paw.
Shaking [11]	Cat rotates its abdomen or head rapidly from side to side.
***Other***	
Other	Any behaviour that does not fit into one the behaviours included in this ethogram.
Out of sight	Cat is not visible to the observer.
Allogrooming [11]	Cat licks the fur on the head or body of another cat.
Human contact	Cat is having direct contact with a human, either being petted or being held/carried.

**Table 2:** Description of identifier variables.

Identifier Variable	Description
Mean	Mean, calculated for every second using the raw acceleration data (30 measures per second).
Sum	Sum, calculated for every second using the raw acceleration data. ${Sum}_{\left(Axis\right)}=\sum_{i=1}^{N}{Axis}_{i}$
Minimum (min)	Minimum value of every 30 measures within each second.
Maximum (max)	Maximum value of every 30 measures within each second.
Standard deviation (sd)	Measures the spread of the data.
Skewness	Asymmetry of the distribution.
Kurtosis	Weight of the tails relative to a normal distribution.
Vector magnitude (VM)	$VM=\sqrt{X^{2}+Y^{2}+Z^{2}}$
Overall dynamic body acceleration (ODBA)	$ODBA=\sum_{i=1}^{N}\left|{DBA}_{X}\right|+\left|{DBA}_{Y}\right|+\left|{DBA}_{Z}\right|$
Dynamic body acceleration (DBA) ^1^	$DBA={Sum}_{axis}-moving\;average$

^1^ Accelerometer data from each axis were individually smoothed using the moving average over 1 s. The DBA was not included as an identifier variable.

**Table 3:** Kappa and overall accuracy values for each modelling round, mounting location (collar and harness), and modelling technique (random forest (RF) and self-organizing map (SOM)).

Modelling Round	Kappa		Overall Accuracy	
	RF	SOM	RF	SOM
	***Collar***			
1	0.642	0.962	0.7	0.968
2	0.678	0.997	0.733	0.997
3	0.684	0.953	0.739	0.961
4	0.742	0.999	0.83	0.999
	***Harness***			
1	0.729	0.962	0.772	0.968
2	0.753	0.996	0.795	0.997
3	0.757	0.997	0.799	0.998
4	0.739	0.999	0.827	0.999

**Table 4:** Differences in mean ± standard error daily percentages of identified behaviours between models for modelling round 1.

	CRF *		HRF *		CSOM *		HSOM *	
**Climbing**	-		0.03 ± 0.01		-		-	
**Jumping**	0.01 ± 0.00		0.03 ± 0.01		-		-	
**Rubbing**	0.07 ± 0.01		0.11 ± 0.04		1.89 ± 0.43		0.42 ± 0.15	
**Trotting**	-		-		-		-	
**Walking ^†^**	2.51 ± 0.33	^b^	3.02 ± 0.38	^b^	0.45 ± 0.07	^a^	4.57 ± 0.58	^b^
**Lying ^†^**	52.69 ± 2.21	^d^	47.54 ± 2.80	^c^	28.20 ± 2.27	^a^	33.76 ± 2.04	^b^
**Sitting ^†^**	25.29 ± 2.45	^c^	28.93 ± 2.88	^c^	23.08 ± 2.79	^b^	18.74 ± 1.89	^a^
**Standing ^†^**	8.08 ± 1.18	^b^	8.16 ± 0.87	^b^	7.70 ± 3.16	^a^	8.83 ± 1.08	^c^
**Grooming ^†^**	6.99 ± 0.29	^a^	7.99 ± 0.31	^a^	15.92 ± 0.75	^b^	20.68 ± 1.50	^c^
**Littering**	0.06 ± 0.01	^a^	0.64 ± 0.25	^b^	-		-	
**Digging**	-		0.01 ± 0.00		-		-	
**Eating ^†^**	4.08 ± 0.45	^b^	3.32 ± 0.30	^a^	22.67 ± 1.56	^c^	12.99 ± 1.30	^c^
**Scratching**	0.20 ± 0.03	^b^	0.21 ± 0.03	^b^	0.08 ± 0.02	^a^	-	
**Shaking**	0.02 ± 0.00		0.01 ± 0.00		-		-	
**Allogrooming**	-		0.01 ± 0.00		-		-	

^a–d^ Different superscripts within a behaviour indicate a significant difference (*p* < 0.05). ^†^ Behaviour was significantly affected by day (*p* < 0.05). * CRF = Collar Random Forest, HRF = Harness Random Forest, CSOM = Collar Self-Organizing Map, HSOM = Harness Self-Organizing Map.

**Table 5:** Differences in mean ± standard error daily percentages of identified behaviours between models for modelling round 2.

	CRF *		HRF *		CSOM *		HSOM *	
**Active ^†^**	2.71 ± 0.37	^c^	3.22 ± 0.41	^c^	0.09 ± 0.01	^a^	2.64 ± 0.27	^b^
**Lying**	52.52 ± 2.37	^d^	47.73 ± 2.92	^c^	29.17 ± 1.85	^a^	38.00 ± 2.39	^b^
**Sitting ^†^**	25.35 ± 2.58	^c^	28.57 ± 3.01	^c^	24.46 ± 3.12	^b^	17.47 ± 1.96	^a^
**Standing ^†^**	7.95 ± 1.25	^b^	8.37 ± 0.93	^b^	3.99 ± 2.85	^a^	11.81 ± 1.29	^b^
**Grooming ^†^**	7.15 ± 0.31	^a^	8.07 ± 0.33	^a^	18.91 ± 0.89	^b^	16.14 ± 1.50	^c^
**Littering**	0.07 ± 0.02		0.62 ± 0.25		-		-	
**Eating ^†^**	4.05 ± 0.47	^b^	3.22 ± 0.31	^a^	22.15 ± 1.68	^d^	13.95 ± 1.30	^c^
**Scratching**	0.19 ± 0.03		0.20 ± 0.03		1.24 ± 0.42		-	

^a–d^ Different superscripts within a behaviour indicate a significant difference (*p* < 0.05). ^†^ Behaviour was significantly affected by day (*p* < 0.05). * CRF = Collar Random Forest, HRF = Harness Random Forest, CSOM = Collar Self-Organizing Map, HSOM = Harness Self-Organizing Map.

**Table 6:** Differences in mean ± standard error daily percentages of identified behaviours between models for modelling round 3.

	CRF *		HRF *		CSOM *		HSOM *	
**Active ^†^**	2.70 ± 0.37	^c^	3.17 ± 0.40	^c^	0.10 ± 0.02	^a^	1.87 ± 0.21	^b^
**Lying ^†^**	53.21 ± 2.34	^c^	49.82 ± 2.85	^c^	27.82 ± 2.98	^a^	39.58 ± 2.00	^b^
**Sitting ^†^**	24.88 ± 2.58	^b^	27.35 ± 3.02	^b^	28.04 ± 3.40	^c^	20.66 ± 2.21	^a^
**Standing ^†^**	7.64 ± 1.30	^b^	7.92 ± 0.97	^b^	3.33 ± 2.76	^a^	9.57 ± 1.00	^c^
**Grooming ^†^**	7.59 ± 0.35	^a^	8.45 ± 0.35	^a^	13.91 ± 0.99	^b^	13.20 ± 1.55	^a^
**Eating ^†^**	3.98 ± 0.47	^a^	3.28 ± 0.31	^a^	26.79 ± 2.91	^c^	14.11 ± 1.47	^b^

^a–c^ Different superscripts within a behaviour indicate a significant difference (*p* < 0.05). ^†^ Behaviour was significantly affected by day (*p* < 0.05). * CRF = Collar Random Forest, HRF = Harness Random Forest, CSOM = Collar Self-Organizing Map, HSOM = Harness Self-Organizing Map.

**Table 7:** Differences in mean ± standard error daily percentages of identified behaviours between models for modelling round 4.

	CRF *		HRF *		CSOM *		HSOM *	
**Active**	4.64 ± 0.49	^a^	5.20 ± 0.61	^a^	14.78 ± 2.09	^b^	10.81 ± 0.72	^b^
**Inactive**	84.46 ± 0.58	^c^	83.27 ± 0.57	^c^	51.16 ± 3.27	^a^	61.98 ± 2.30	^b^
**Maintenance**	10.90 ± 0.47	^a^	11.53 ± 0.46	^a^	34.06 ± 1.59	^b^	27.21 ± 2.35	^b^

^a–c^ Different superscripts within a behaviour indicate a significant difference (*p* < 0.05). * CRF = Collar Random Forest, HRF = Harness Random Forest, CSOM = Collar Self-Organizing Map, HSOM = Harness Self-Organizing Map.

**Figure A1:**
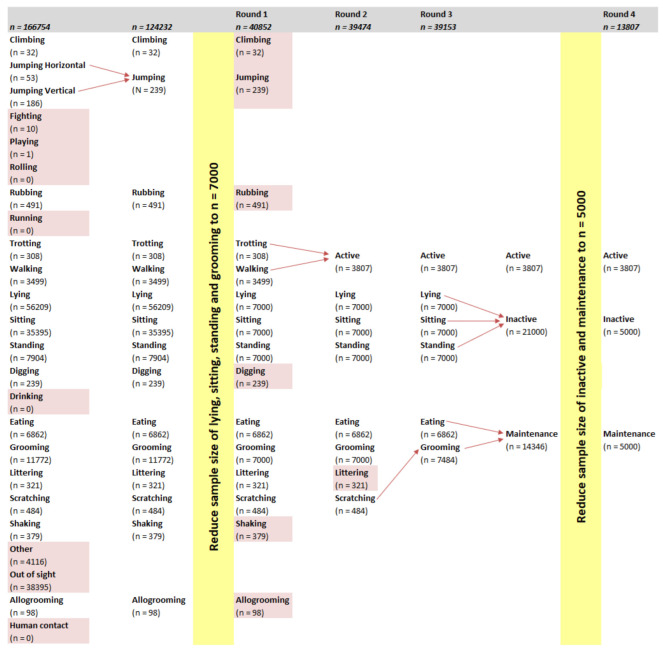
Process of behavioural selection before and between modelling building rounds. Behaviours marked in red were removed. Red arrows indicate the combining of behaviours into new or existing categories. The first two columns show the deletion and merging of behaviours before the first modelling round. The number of observations per behaviour is stated in brackets under the behaviour (n in seconds), and the total number (in seconds) of all behavioural observations is at the top of each column.

## Data Availability

R-dataframes of the data presented in this study are openly available in FigShare at https://doi.org/10.6084/m9.figshare.23605842 (accessed on 30 June 2023) [41]. Raw datafiles are available on request from the corresponding author due to the large size. R-scripts are openly available through GitHub [18].

## Supplementary Materials

The following supporting information can be downloaded at: https://www.mdpi.com/article/10.3390/s23167165/s1, Supplementary Material S1: Confusion matrices of identification models; Supplementary Material S2: Accuracies of identification models; Supplementary Material S3: Precisions of identification models; Supplementary Material S4: Sensitivities of identification models; Supplementary Material S5: Specificities of identification models.
