# Supplementary material for: The Use of Triaxial Accelerometers and Machine Learning Algorithms for Behavioural Identification in Domestic Cats (Felis catus): A Validation Study
Source: Sensors (Basel). 2023 Aug 14;23(16):7165. doi: 10.3390/s23167165 (PMC10458840; doi:10.3390/s23167165)
Supplement: Supplementary file 1 [file sensors-23-07165-s001.zip › sensors-2506955-supplementary/S1 - Confusion Matrices.pdf]

Supplementary Material 1 – Confusion matrices of identification models

Table S1. Confusion matrix for modelling round 1 of the collar-mounted data using a Random Forest model. Numbers along the diagonal represent the true positive (TP) value. Pred = identified behaviour, Ob = observed behaviour.

Pred \ Ob	Climbing	Jumping	Rubbing	Trotting	Walking	Lying	Sitting	Standing	Grooming	Littering	Digging	Eating	Scratching	Shaking	Allogrooming
**Climbing**	0	0	0	0	0	0	0	0	0	0	0	0	0	0	0
**Jumping**	0	4	0	0	1	0	0	0	0	0	0	0	0	0	0
**Rubbing**	0	0	20	0	1	0	0	0	0	0	0	0	1	2	0
**Trotting**	0	0	0	2	1	0	1	0	0	0	0	0	0	0	0
**Walking**	2	33	23	40	533	0	20	213	43	2	8	41	19	31	1
**Lying**	0	0	5	0	9	1855	203	98	39	10	0	8	2	6	1
**Sitting**	0	3	3	5	30	139	1536	161	70	26	0	9	2	5	1
**Standing**	3	17	17	28	226	80	233	1231	152	18	4	86	25	28	3
**Grooming**	4	6	46	14	156	12	77	212	1526	4	17	137	26	29	19
**Littering**	0	0	0	0	0	2	3	2	0	34	0	0	0	0	0
**Digging**	0	0	0	0	0	0	0	0	0	0	0	0	0	0	0
**Eating**	0	6	33	3	81	12	23	174	269	2	42	1776	3	4	4
**Scratching**	0	1	0	0	7	0	4	2	1	0	0	1	67	2	0
**Shaking**	0	1	0	0	4	0	0	7	0	0	0	0	0	6	0
**Allogrooming**	0	0	0	0	0	0	0	0	0	0	0	0	0	0	0

Table S2. Confusion matrix for modelling round 1 of the harness-mounted data using a Random Forest model. Numbers along the diagonal represent the true positive (TP) value. Pred = identified behaviour, Ob = observed behaviour.

Pred \ Ob	Climbing	Jumping	Rubbing	Trotting	Walking	Lying	Sitting	Standing	Grooming	Littering	Digging	Eating	Scratching	Shaking	Allogrooming
**Climbing**	1	0	0	0	2	0	0	1	0	0	0	0	0	0	0
**Jumping**	1	4	0	0	4	0	0	1	0	0	0	2	0	2	0
**Rubbing**	0	1	70	2	13	1	1	25	11	0	3	2	1	3	0
**Trotting**	0	0	0	2	1	0	1	0	0	0	0	0	0	0	0
**Walking**	6	38	17	38	680	2	11	266	25	6	16	23	26	36	1
**Lying**	0	0	7	0	4	1989	27	73	9	6	0	10	0	4	0
**Sitting**	0	5	2	7	33	13	1711	138	91	10	0	0	0	9	5
**Standing**	0	7	12	37	187	64	232	1226	137	35	9	64	18	27	6
**Grooming**	1	9	27	6	90	21	107	254	1761	2	6	35	34	23	13
**Littering**	0	0	0	0	0	0	3	5	0	34	0	0	0	0	0
**Digging**	0	0	0	0	0	0	0	0	1	1	10	2	0	0	0
**Eating**	0	7	12	0	32	10	4	110	65	2	27	1920	1	4	2
**Scratching**	0	0	0	0	3	0	3	1	0	0	0	0	65	2	0
**Shaking**	0	0	0	0	0	0	0	0	0	0	0	0	0	3	0
**Allogrooming**	0	0	0	0	0	0	0	0	0	0	0	0	0	0	2

Table S3. Confusion matrix for modelling round 1 of the collar-mounted data using a Self-Organizing Map model. Numbers along the diagonal represent the true positive (TP) value. Pred = identified behaviour, Ob = observed behaviour.

Ob<br>Pred	Climbing	Jumping	Rubbing	Trotting	Walking	Lying	Sitting	Standing	Grooming	Littering	Digging	Eating	Scratching	Shaking	Allogrooming
**Climbing**	0	0	0	0	0	0	0	0	0	0	0	0	0	0	0
**Jumping**	0	0	0	0	0	0	0	0	0	0	0	0	0	0	0
**Rubbing**	3	17	90	16	0	0	0	0	0	11	43	0	0	12	6
**Trotting**	2	29	36	65	0	0	0	0	0	2	25	0	28	40	15
**Walking**	0	3	1	1	1049	0	5	11	2	0	0	1	1	17	0
**Lying**	0	0	0	0	0	2100	0	0	0	0	0	0	0	0	0
**Sitting**	0	0	0	0	0	0	2095	0	0	0	0	0	0	0	0
**Standing**	1	0	1	0	0	0	0	2089	0	0	0	0	0	0	0
**Grooming**	1	2	3	0	0	0	0	0	2098	0	0	0	1	0	0
**Littering**	0	0	0	0	0	0	0	0	0	83	0	0	0	0	0
**Digging**	0	0	0	0	0	0	0	0	0	0	0	0	0	0	0
**Eating**	0	0	0	0	0	0	0	0	0	0	0	2057	0	0	0
**Scratching**	0	0	0	0	0	0	0	0	0	0	0	0	115	0	0
**Shaking**	2	20	16	10	0	0	0	0	0	0	3	0	0	44	8
**Allogrooming**	0	0	0	0	0	0	0	0	0	0	0	0	0	0	0

Table S4. Confusion matrix for modelling round 1 of the harness-mounted data using a Self-Organizing Map model. Numbers along the diagonal represent the true positive (TP) value. Pred = identified behaviour, Ob = observed behaviour.

Ob<br>Pred	Climbing	Jumping	Rubbing	Trotting	Walking	Lying	Sitting	Standing	Grooming	Littering	Digging	Eating	Scratching	Shaking	Allogrooming
**Climbing**	0	0	0	0	0	0	0	0	0	0	0	0	0	0	0
**Jumping**	0	0	0	0	0	0	0	0	0	0	0	0	0	0	0
**Rubbing**	0	0	145	0	0	0	0	0	0	0	0	0	0	0	0
**Trotting**	0	9	0	40	0	0	1	0	0	14	0	0	14	9	1
**Walking**	0	0	0	1	1039	0	2	16	1	0	0	1	1	11	0
**Lying**	0	0	1	0	1	2100	0	0	0	0	0	0	0	0	0
**Sitting**	0	0	0	1	0	0	2097	0	0	0	0	0	0	0	0
**Standing**	0	0	0	2	9	0	0	2084	1	0	0	1	2	5	0
**Grooming**	0	0	0	0	0	0	0	0	2098	0	0	0	5	0	0
**Littering**	0	4	0	4	0	0	0	0	0	52	1	0	30	16	25
**Digging**	0	0	0	0	0	0	0	0	0	0	68	0	0	0	0
**Eating**	0	3	0	1	0	0	0	0	0	0	0	2055	0	3	0
**Scratching**	9	19	1	24	0	0	0	0	0	23	2	1	76	37	1
**Shaking**	0	36	0	19	0	0	0	0	0	7	0	0	17	32	2
**Allogrooming**	0	0	0	0	0	0	0	0	0	0	0	0	0	0	0

Table S5. Confusion matrix for modelling round 2 of the collar data using a Random Forest model. Numbers along the diagonal represent the true positive (TP) value. Pred = identified behaviour, Ob = observed behaviour.

Pred \ Ob	Active	Lying	Sitting	Standing	Grooming	Littering	Eating	Scratching
Active	596	2	12	231	45	4	38	20
Lying	13	1861	187	106	41	8	8	3
Sitting	36	125	1554	159	75	19	5	3
Standing	220	85	235	1226	122	18	83	19
Grooming	164	11	84	220	1573	3	140	34
Littering	0	1	3	3	0	36	0	0
Eating	107	15	24	152	272	8	1784	1
Scratching	6	0	1	3	1	0	0	65

Table S6. Confusion matrix for modelling round 2 of the harness data using a Random Forest model. Numbers along the diagonal represent the true positive (TP) value. Pred = identified behaviour, Ob = observed behaviour.

Pred \ Ob	Active	Lying	Sitting	Standing	Grooming	Littering	Eating	Scratching
Active	730	3	21	339	35	9	26	29
Lying	20	2007	25	54	10	7	17	1
Sitting	32	12	1690	112	76	11	0	1
Standing	229	59	241	1228	159	22	76	17
Grooming	99	15	117	265	1784	6	46	38
Littering	0	1	3	6	0	40	0	0
Eating	29	3	3	96	61	1	1893	0
Scratching	3	0	0	0	4	0	0	59

Table S7. Confusion matrix for modelling round 2 of the collar data using a Self-Organizing Map model. Numbers along the diagonal represent the true positive (TP) value. Pred = identified behaviour, Ob = observed behaviour.

Pred \ Ob	Active	Lying	Sitting	Standing	Grooming	Littering	Eating	Scratching
Active	1142	0	3	13	2	1	0	4
Lying	0	2100	0	0	0	0	0	3
Sitting	0	0	2097	0	0	0	0	2
Standing	0	0	0	2087	0	0	0	2
Grooming	0	0	0	0	2127	0	0	3
Littering	0	0	0	0	0	94	0	0
Eating	0	0	0	0	0	1	2058	0
Scratching	0	0	0	0	0	0	0	131

Table S8. Confusion matrix for modelling round 2 of the harness data using a Self-Organizing Map model. Numbers along the diagonal represent the true positive (TP) value. Pred = identified behaviour, Ob = observed behaviour.

Pred \ Ob	Active	Lying	Sitting	Standing	Grooming	Littering	Eating	Scratching
Active	1128	0	0	11	0	0	2	1
Lying	0	2100	0	0	0	0	0	0
Sitting	0	0	2099	0	0	0	0	1
Standing	14	0	1	2089	1	0	0	0
Grooming	0	0	0	0	2128	0	0	7
Littering	0	0	0	0	0	96	0	0
Eating	0	0	0	0	0	0	2056	0
Scratching	0	0	0	0	0	0	0	136

Table S9. Confusion matrix for modelling round 3 of the collar data using a Random Forest model. Numbers along the diagonal represent the true positive (TP) value. Pred = identified behaviour, Ob = observed behaviour.

Pred \ Ob	Active	Lying	Sitting	Standing	Grooming	Eating
Active	615	1	17	240	66	45
Lying	17	1870	223	112	44	10
Sitting	44	110	1543	174	64	9
Standing	205	91	234	1175	151	79
Grooming	191	12	66	241	1709	129
Eating	70	16	17	158	240	1786

Table S10. Confusion matrix for modelling round 3 of the harness data using a Random Forest model. Numbers along the diagonal represent the true positive (TP) value. Pred = identified behaviour, Ob = observed behaviour.

Pred \ Ob	Active	Lying	Sitting	Standing	Grooming	Eating
**Active**	745	2	21	328	50	26
**Lying**	9	1997	33	70	14	10
**Sitting**	32	17	1648	127	75	0
**Standing**	214	59	274	1209	173	79
**Grooming**	103	20	120	252	1898	37
**Eating**	39	5	4	114	64	1906

Table S11. Confusion matrix for modelling round 3 of the collar data using a Self-Organizing Map model. Numbers along the diagonal represent the true positive (TP) value. Pred = identified behaviour, Ob = observed behaviour.

Pred \ Ob	Active	Lying	Sitting	Standing	Grooming	Eating
**Active**	1006	1	4	15	2	0
**Lying**	0	1964	0	0	0	0
**Sitting**	0	0	2093	0	0	0
**Standing**	0	1	0	2077	0	0
**Grooming**	0	0	0	0	2118	0
**Eating**	136	134	3	8	154	2058

Table S12. Confusion matrix for modelling round 3 of the harness data using a Self-Organizing Map model. Numbers along the diagonal represent the true positive (TP) value. Pred = identified behaviour, Ob = observed behaviour.

Pred \ Ob	Active	Lying	Sitting	Standing	Grooming	Eating
**Active**	1137	0	3	17	1	0
**Lying**	0	2100	0	0	0	0
**Sitting**	1	0	2097	0	0	0
**Standing**	0	0	0	2081	0	0
**Grooming**	0	0	0	0	2273	0
**Eating**	4	0	0	2	0	2058

Table S13. Confusion matrix for modelling round 3 of the collar data using a Random Forest model. Numbers along the diagonal represent the true positive (TP) value. Pred = identified behaviour, Ob = observed behaviour.

Pred \ Ob	Active	Inactive	Maintenance
**Active**	905	147	69
**Inactive**	125	1211	111
**Maintenance**	112	142	1320

Table S14. Confusion matrix for modelling round 3 of the harness data using a Random Forest model. Numbers along the diagonal represent the true positive (TP) value. Pred = identified behaviour, Ob = observed behaviour.

Pred \ Ob	Active	Inactive	Maintenance
**Active**	901	158	85
**Inactive**	112	1196	86
**Maintenance**	129	146	1329

Table S15. Confusion matrix for modelling round 3 of the collar data using a Self-Organizing Map model. Numbers along the diagonal represent the true positive (TP) value. Pred = identified behaviour, Ob = observed behaviour.

Pred \ Ob	Active	Inactive	Maintenance
**Active**	1142	1	2
**Inactive**	0	1499	0
**Maintenance**	0	0	1498

Table S16. Confusion matrix for modelling round 3 of the harness data using a Self-Organizing Map model. Numbers along the diagonal represent the true positive (TP) value. Pred = identified behaviour, Ob = observed behaviour.

Pred \ Ob	Active	Inactive	Maintenance
**Active**	1142	2	1
**Inactive**	0	1497	0
**Maintenance**	0	1	1499
