# Supplementary material for: The Use of Triaxial Accelerometers and Machine Learning Algorithms for Behavioural Identification in Domestic Cats (Felis catus): A Validation Study
Source: Sensors (Basel). 2023 Aug 14;23(16):7165. doi: 10.3390/s23167165 (PMC10458840; doi:10.3390/s23167165)
Supplement: Supplementary file 1 [file sensors-23-07165-s001.zip › sensors-2506955-supplementary/S2 - Accuracy of models.pdf]

Supplementary Material 2 – Accuracies of identification models

Table S17. Accuracy for modelling round 1. CRF = Collar Random Forest, HRF = Harness Random Forest, CSOM = Collar Self-Organizing Map, HSOM = Harness Self-Organizing Map.

	CRF	HRF	CSOM	HSOM
Climbing	0.999	0.999	0.999	0.999
Jumping	0.994	0.994	0.994	0.994
Rubbing	0.989	0.989	0.987	1.000
Trotting	0.992	0.992	0.983	0.992
Walking	0.919	0.928	0.997	0.996
Lying	0.949	0.980	1.000	1.000
Sitting	0.917	0.943	1.000	1.000
Standing	0.854	0.861	0.999	0.997
Grooming	0.891	0.921	0.999	0.999
Littering	0.994	0.994	0.999	0.990
Digging	0.994	0.995	0.994	1.000
Eating	0.923	0.966	1.000	0.999
Scratching	0.992	0.993	0.998	0.985
Shaking	0.990	0.991	0.990	0.987
Allogrooming	0.998	0.998	0.998	0.998

Table S18. Accuracy for modelling round 2. CRF = Collar Random Forest, HRF = Harness Random Forest, CSOM = Collar Self-Organizing Map, HSOM = Harness Self-Organizing Map.

	CRF	HRF	CSOM	HSOM
Active	0.924	0.926	0.998	0.998
Lying	0.949	0.981	1.000	1.000
Sitting	0.918	0.945	1.000	1.000
Standing	0.860	0.859	0.999	0.998
Grooming	0.898	0.922	1.000	0.999
Littering	0.994	0.994	1.000	1.000
Eating	0.928	0.970	1.000	1.000
Scratching	0.992	0.992	0.999	0.999

Table S19. Accuracy for modelling round 3. CRF = Collar Random Forest, HRF = Harness Random Forest, CSOM = Collar Self-Organizing Map, HSOM = Harness Self-Organizing Map.

	CRF	HRF	CSOM	HSOM
Active	0.924	0.930	0.987	0.998
Lying	0.946	0.980	0.988	1.000
Sitting	0.919	0.940	0.999	1.000
Standing	0.857	0.856	0.998	0.998
Grooming	0.903	0.948	0.987	1.000
Eating	0.746	0.790	0.963	0.999

Table S20. Accuracy for modelling round 4. CRF = Collar Random Forest, HRF = Harness Random Forest, CSOM = Collar Self-Organizing Map, HSOM = Harness Self-Organizing Map.

	CRF	HRF	CSOM	HSOM
Active	0.891	0.883	0.999	0.999
Inactive	0.873	0.879	1.000	0.999
Maintenance	0.895	0.892	1.000	1.000
