# Supplementary material for: The Use of Triaxial Accelerometers and Machine Learning Algorithms for Behavioural Identification in Domestic Cats (Felis catus): A Validation Study
Source: Sensors (Basel). 2023 Aug 14;23(16):7165. doi: 10.3390/s23167165 (PMC10458840; doi:10.3390/s23167165)
Supplement: Supplementary file 1 [file sensors-23-07165-s001.zip › sensors-2506955-supplementary/S3 - Precision of models.pdf]

Supplementary Material 3 – Precisions of identification models

Table S21. Precision for modelling round 1. CRF = Collar Random Forest, HRF = Harness Random Forest, CSOM = Collar Self-Organizing Map, HSOM = Harness Self-Organizing Map, N/A = not available.

	CRF	HRF	CSOM	HSOM
**Climbing**	N/A	0.250	N/A	N/A
**Jumping**	0.800	0.286	N/A	N/A
**Rubbing**	0.833	0.526	0.455	1.000
**Trotting**	0.500	0.500	0.269	0.455
**Walking**	0.528	0.571	0.962	0.969
**Lying**	0.830	0.934	1.000	0.999
**Sitting**	0.772	0.845	1.000	1.000
**Standing**	0.572	0.595	0.999	0.990
**Grooming**	0.668	0.737	0.997	0.998
**Littering**	0.829	0.810	1.000	0.394
**Digging**	N/A	0.714	N/A	1.000
**Eating**	0.730	0.874	1.000	0.997
**Scratching**	0.788	0.878	1.000	0.394
**Shaking**	0.333	1.000	0.427	0.283
**Allogrooming**	N/A	1.000	N/A	N/A

Table S22. Precision for modelling round 2. CRF = Collar Random Forest, HRF = Harness Random Forest, CSOM = Collar Self-Organizing Map, HSOM = Harness Self-Organizing Map, N/A = not available.

	CRF	HRF	CSOM	HSOM
**Active**	0.629	0.612	0.980	0.988
**Lying**	0.836	0.937	0.999	1.000
**Sitting**	0.786	0.874	0.999	1.000
**Standing**	0.611	0.605	0.999	0.992
**Grooming**	0.706	0.753	0.999	0.997
**Littering**	0.837	0.800	1.000	1.000
**Eating**	0.755	0.907	1.000	1.000
**Scratching**	0.855	0.894	1.000	1.000

Table S23. Precision for modelling round 3. CRF = Collar Random Forest, HRF = Harness Random Forest, CSOM = Collar Self-Organizing Map, HSOM = Harness Self-Organizing Map.

	CRF	HRF	CSOM	HSOM
**Active**	0.625	0.636	0.979	0.982
**Lying**	0.822	0.936	1.000	1.000
**Sitting**	0.794	0.868	1.000	1.000
**Standing**	0.607	0.602	1.000	1.000
**Grooming**	0.747	0.890	1.000	1.000
**Eating**	0.397	0.451	0.826	0.997

Table S24. Precision for modelling round 3. CRF = Collar Random Forest, HRF = Harness Random Forest, CSOM = Collar Self-Organizing Map, HSOM = Harness Self-Organizing Map.

	CRF	HRF	CSOM	HSOM
**Active**	0.807	0.788	0.997	0.997
**Inactive**	0.837	0.858	1.000	1.000
**Maintenance**	0.839	0.829	1.000	0.999
