# Supplementary material for: The Use of Triaxial Accelerometers and Machine Learning Algorithms for Behavioural Identification in Domestic Cats (Felis catus): A Validation Study
Source: Sensors (Basel). 2023 Aug 14;23(16):7165. doi: 10.3390/s23167165 (PMC10458840; doi:10.3390/s23167165)
Supplement: Supplementary file 1 [file sensors-23-07165-s001.zip › sensors-2506955-supplementary/S4 - Sensitivity of models.pdf]

Supplementary Material 4 – Sensitivities of identification models

Table S25. Sensitivity for modelling round 1. CRF = Collar Random Forest, HRF = Harness Random Forest, CSOM = Collar Self-Organizing Map, HSOM = Harness Self-Organizing Map.

	CRF	HRF	CSOM	HSOM
**Climbing**	0.000	0.111	0.000	0.000
**Jumping**	0.056	0.056	0.000	0.000
**Rubbing**	0.136	0.476	0.612	0.986
**Trotting**	0.022	0.022	0.707	0.435
**Walking**	0.508	0.648	1.000	0.990
**Lying**	0.883	0.947	1.000	1.000
**Sitting**	0.731	0.815	0.998	0.999
**Standing**	0.586	0.584	0.995	0.992
**Grooming**	0.727	0.839	0.999	0.999
**Littering**	0.354	0.354	0.865	0.542
**Digging**	0.000	0.141	0.000	0.958
**Eating**	0.863	0.933	1.000	0.999
**Scratching**	0.462	0.448	0.793	0.524
**Shaking**	0.053	0.027	0.389	0.283
**Allogrooming**	0.000	0.069	0.000	0.000

Table S26. Sensitivity for modelling round 2. CRF = Collar Random Forest, HRF = Harness Random Forest, CSOM = Collar Self-Organizing Map, HSOM = Harness Self-Organizing Map.

	CRF	HRF	CSOM	HSOM
**Active**	0.522	0.639	1.000	0.988
**Lying**	0.886	0.956	1.000	1.000
**Sitting**	0.740	0.805	0.999	1.000
**Standing**	0.584	0.585	0.994	0.995
**Grooming**	0.739	0.838	0.999	1.000
**Littering**	0.375	0.417	0.979	1.000
**Eating**	0.867	0.920	1.000	0.999
**Scratching**	0.448	0.407	0.903	0.938

Table S27. Sensitivity for modelling round 3. CRF = Collar Random Forest, HRF = Harness Random Forest, CSOM = Collar Self-Organizing Map, HSOM = Harness Self-Organizing Map.

	CRF	HRF	CSOM	HSOM
**Active**	0.539	0.652	0.881	0.996
**Lying**	0.890	0.951	0.935	1.000
**Sitting**	0.735	0.785	0.997	0.999
**Standing**	0.560	0.576	0.989	0.991
**Grooming**	0.752	0.835	0.931	1.000
**Eating**	0.868	0.926	1.000	1.000

Table S28. Sensitivity for modelling round 4. CRF = Collar Random Forest, HRF = Harness Random Forest, CSOM = Collar Self-Organizing Map, HSOM = Harness Self-Organizing Map.

	CRF	HRF	CSOM	HSOM
**Active**	0.807	0.788	0.997	0.997
**Inactive**	0.837	0.858	1.000	1.000
**Maintenance**	0.839	0.829	1.000	0.999
