# Supplementary material for: The Use of Triaxial Accelerometers and Machine Learning Algorithms for Behavioural Identification in Domestic Cats (Felis catus): A Validation Study
Source: Sensors (Basel). 2023 Aug 14;23(16):7165. doi: 10.3390/s23167165 (PMC10458840; doi:10.3390/s23167165)
Supplement: Supplementary file 1 [file sensors-23-07165-s001.zip › sensors-2506955-supplementary/S5 - Specificity of models.pdf]

Supplementary Material 5 – Specificities of identification models

Table S29. Specificity for modelling round 1. CRF = Collar Random Forest, HRF = Harness Random Forest, CSOM = Collar Self-Organizing Map, HSOM = Harness Self-Organizing Map.

	CRF	HRF	CSOM	HSOM
**Climbing**	1.000	1.000	1.000	1.000
**Jumping**	1.000	0.999	1.000	1.000
**Rubbing**	1.000	0.995	0.991	1.000
**Trotting**	1.000	1.000	0.985	0.996
**Walking**	0.958	0.954	0.996	0.997
**Lying**	0.962	0.986	1.000	1.000
**Sitting**	0.955	0.969	1.000	1.000
**Standing**	0.909	0.918	1.000	0.998
**Grooming**	0.925	0.938	0.999	1.000
**Littering**	0.999	0.999	1.000	0.993
**Digging**	1.000	1.000	1.000	1.000
**Eating**	0.936	0.973	1.000	0.999
**Scratching**	0.999	0.999	1.000	0.990
**Shaking**	0.999	1.000	0.995	0.993
**Allogrooming**	1.000	1.000	1.000	1.000

Table S30. Specificity for modelling round 2. CRF = Collar Random Forest, HRF = Harness Random Forest, CSOM = Collar Self-Organizing Map, HSOM = Harness Self-Organizing Map.

	CRF	HRF	CSOM	HSOM
**Active**	0.967	0.957	0.998	0.999
**Lying**	0.963	0.986	1.000	1.000
**Sitting**	0.957	0.975	1.000	1.000
**Standing**	0.920	0.918	1.000	0.998
**Grooming**	0.933	0.940	1.000	0.999
**Littering**	0.999	0.999	1.000	1.000
**Eating**	0.941	0.980	1.000	1.000
**Scratching**	0.999	0.999	1.000	1.000

Table S31. Specificity for modelling round 3. CRF = Collar Random Forest, HRF = Harness Random Forest, CSOM = Collar Self-Organizing Map, HSOM = Harness Self-Organizing Map.

	CRF	HRF	CSOM	HSOM
**Active**	0.965	0.960	0.998	0.998
**Lying**	0.958	0.986	1.000	1.000
**Sitting**	0.959	0.974	1.000	1.000
**Standing**	0.921	0.917	1.000	1.000
**Grooming**	0.939	0.975	1.000	1.000
**Eating**	0.720	0.761	0.955	0.999

Table S32. Specificity for modelling round 4. CRF = Collar Random Forest, HRF = Harness Random Forest, CSOM = Collar Self-Organizing Map, HSOM = Harness Self-Organizing Map.

	CRF	HRF	CSOM	HSOM
**Active**	0.928	0.919	0.999	0.999
**Inactive**	0.911	0.925	1.000	1.000
**Maintenance**	0.904	0.896	1.000	1.000
